# Determinants for scalable adoption of autonomous AI in the detection of diabetic eye disease in diverse practice types: key best practices learned through collection of real-world data

**DOI:** 10.3389/fdgth.2023.1004130

**Published:** 2023-05-18

**Authors:** Juli Goldstein, Dena Weitzman, Meghan Lemerond, Andrew Jones

**Affiliations:** Digital Diagnostics Inc., Coralville, IA, United States

**Keywords:** clinical workflow, artificial intelligence, diabetic eye disease, improving access, diabetes, autonomous artificial intelligence

## Abstract

Autonomous Artificial Intelligence (AI) has the potential to reduce disparities, improve quality of care, and reduce cost by improving access to specialty diagnoses at the point-of-care. Diabetes and related complications represent a significant source of health disparities. Vision loss is a complication of diabetes, and there is extensive evidence supporting annual eye exams for prevention. Prior to the use of autonomous AI, store-and-forward imaging approaches using remote reading centers (asynchronous telemedicine) attempted to increase diabetes related eye exams with limited success. In 2018, after rigorous clinical validation, the first fully autonomous AI system [LumineticsCore™ (formerly IDx-DR), Digital Diagnostics Inc., Coralville, IA, United States] received U.S. Food and Drug Administration (FDA) *De Novo* authorization. The system diagnoses diabetic retinopathy (including macular edema) without specialist physician overread at the point-of-care. In addition to regulatory clearance, reimbursement, and quality measure updates, successful adoption requires local optimization of the clinical workflow. The general challenges of frontline care clinical workflow have been well documented in the literature. Because healthcare AI is so new, there remains a gap in the literature about challenges and opportunities to embed diagnostic AI into the clinical workflow. The goal of this review is to identify common workflow themes leading to successful adoption, measured as attainment number of exams per month using the autonomous AI system against targets set for each health center. We characterized the workflow in four different US health centers over a 12-month period. Health centers were geographically dispersed across the Midwest, Southwest, Northeast, and West Coast and varied distinctly in terms of size, staffing, resources, financing and demographics of patient populations. After 1 year, the aggregated number of diabetes-related exams per month increased from 89 after the first month of initial deployment to 174 across all sites. Across the diverse practice types, three primary determinants underscored sustainable adoption: (1) Inclusion of Executive and Clinical Champions; (2) Underlining Health Center Resources; and (3) Clinical workflows that contemplate patient identification (pre-visit), LumineticsCore Exam Capture and Provider Consult (patient visit), and Timely Referral Triage (post-visit). In addition to regulatory clearance, reimbursement and quality measures, our review shows that addressing the core determinants for workflow optimization is an essential part of large-scale adoption of innovation. These best practices can be generalizable to other autonomous AI systems in front-line care settings, thereby increasing patient access, improving quality of care, and addressing health disparities.

## Introduction

Autonomous artificial intelligence (AI) diagnostic systems can reduce disparities by improving access to specialty diagnoses at the point-of-care. Much has been published about the responsible adoption of healthcare AI so that it benefits patient outcomes and is shown to be safe and effective, mitigates potential bias, and is developed under an ethical framework (1–3). As described elsewhere, adoption of these new technologies requires meeting the expectations of multiple stakeholders and, at the same time, seamlessly integrating into workflow in the clinical setting (4). Stakeholder considerations for scalable adoption include varying perspectives: for health center staff, ease-of-use and seamless integration with existing workflow; for providers, acquisition costs, patient benefit, and payer reimbursement; for payers, utilization, costs of delivering the service, patient benefit, and cost-savings over standard-of-care; and for regulators, proven safety and efficacy in the targeted population, elimination of potential bias, and adherence to ethical frameworks in the Total Product Lifecycle (TPLC) (5) for AI systems in healthcare (2, 3, 6).

Primary care workflows commonly vary by practice, and at times, for individual providers (4, 7, 8). Furthermore, integration of a new technology into clinical workflow has equity implications related to intended use and need to be addressed early in the design phase of the product development lifecycle (6). The clinical workflow itself, including the demands on clinical labor and healthcare resources, can impact the reimbursement framework (9, 10) and ultimately large-scale adoption. We reviewed the deployment of the first ever fully autonomous AI system to receive FDA clearance (11) and payment on the Medicare Physician Fee Schedule (MPFS) (12) across four distinct health systems with varying resources and patient populations over a 12-month time period.

The learnings from real-world implementation of the autonomous AI system in diverse practice settings allowed us to uncover best practices in clinical workflow and front-line resources for successful adoption of AI technology in front-line settings.

## Autonomous AI for the detection of diabetic retinopathy

Over thirty million people, 10.5% of the US population, have diagnosed diabetes (13). The percentage of adults with diabetes increases with age, reaching almost one out of every three people over the age of 65 years. Ophthalmic complications in people living with diabetes that lead to vision loss are common, and leading societies recommend annual diabetic eye exams to prevent disease progression (14, 15). Diabetes related vision loss is even more pronounced in areas with limited access to specialty care and in vulnerable populations with limited resources (16, 17).

The use of store-and-forward remote reading networks (asynchronous telemedicine) have attempted to move the needle in diabetic eye exam rates with varying success. LumineticsCore™ (formerly IDx-DR) (Digital Diagnostics Inc., Coralville, IA, United States) is an autonomous AI system that was granted *De Novo* authorization by the FDA to diagnose diabetic retinopathy (including diabetic macular edema) in adults living with diabetes after rigorous FDA validation for safety and equity (11). FDA determined that LumineticsCore met the standards for “breakthrough device” designation in accordance with section 3051 of the 21st Century Cures Act.

LumineticsCore provides a result at the point-of-care, without the need for a specialist or remote reading network to interpret the image, transmits results directly into the electronic health record (EHR) where a health system is integrated, and is developed based on a user-centered designed interface so that it can be operated by existing non-physician, clinical staff in multiple care settings, including primary care and internal medicine. The service includes an auto-aligning robotic camera, provides quality control analysis to guide the operator, and assumes liability arising from system failure or misdiagnosis. The AI driven system performs the process of the diabetic eye exam at the point-of-care following similar cognitive processes as a highly trained eye care provider. The entire system design was based on a rigorous, ethical framework for designing, developing, and deploying AI (1–3).

## Deployment of fully autonomous AI across four distinct health systems in distinct geographies with varying size, staffing, resources, finances and demographics of patients served

LumineticsCore systems were fully deployed in various health centers dispersed across the Midwest, Southwest, Northeast, and West Coast that serve adult patients with diabetes. Each deployment included training for operators and health care providers at time of implementation and thereafter as needed. Key performance indicators (KPIs) were set at each individual health center to identify targeted number of eye exams per month. Utilization data was tracked through a Digital Diagnostics' internal platform system, and performance reports were shared with health centers as part of the post-implementation process.

Only one of the healthcare systems had integrated eye specialty care and a clinical quality improvement team. Three of the four health centers placed the autonomous AI system in the primary care and endocrinology locations, whereas one of the larger health centers placed the systems in the centralized location of the lab and endocrinology. The smallest of the four centers had limited resources, with no diabetes educators, or population health team support. See Table 1 for an illustrative description of the different settings.

**Table 1:** Description of health centers observed.

Healthcare center identifier	Center type	Exam setting	Eyecare part of health system	Care team
Health center A	Not-for-profit community-based health system in the Midwest	Endocrinology & lab	No	• RN navigators • Endocrinologists* • Laboratory assistants • Clinic managers • Director diabetes strategy**
Health center B	Regional integrated delivery network in the Southwest	Primary care	No	• RN diabetes navigators • Clinical lead* • Clinic manager director of primary care**
Health center C	Large community-based integrated health system in the Northeast	Endocrinology	Yes	• Clinic managers • Medical director* • Population health/quality team • Ophthalmology**
Health center D	Small group of independent primary practices in the West Coast	Primary care	No	• Clinic manager • Chief medical officer***

* Clinical champion.

** Executive champion.

*** Both clinical and executive champion.

**Figure 1:**
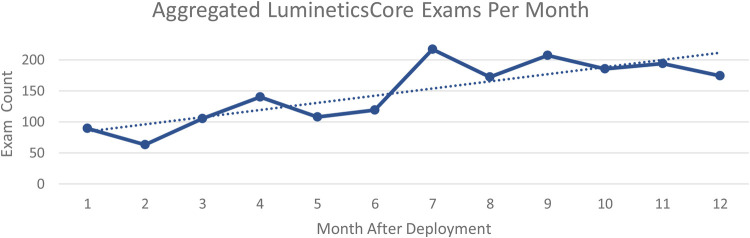
Aggregated LumineticsCore exams per month beginning at initial deployment to 1 year after.

After 1 year, the aggregated number of exams increased from 89 per month at initial deployment to 174 per month across all sites. Figure 1 shows aggregated health center exam performance 12-months after initial launch. The trend line shows the sustainable and ongoing success of implementation of LumineticsCore.

## Determinants: three common themes underscored successful adoption and utilization of fully autonomous AI in the healthcare setting

**Figure 2:**
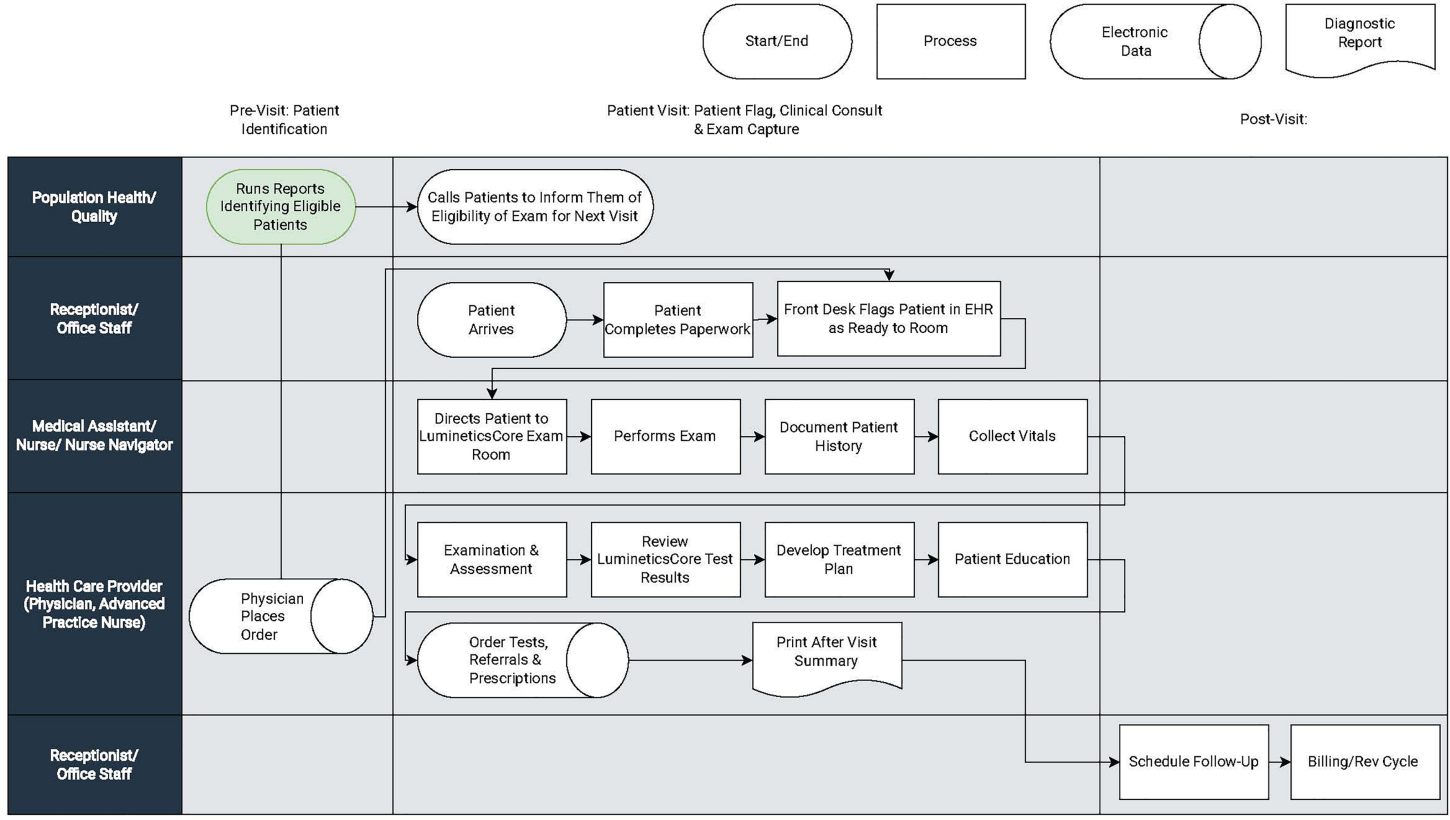
Illustrative sample clinical workflow based on high resource health centers.

Among diverse practice settings, we uncovered three primary determinants in the clinical setting that underscored whether adoption would be successful and scalable: (1) Inclusion of Executive and Clinical Champions; (2) Underlining Health Center Resources; and (3) Clinical workflows that contemplate patient identification (pre-visit), LumineticsCore Exam Capture and Provider Consult (patient visit), and Timely Referral Triage (post-visit).

(I) Inclusion of Executive and Clinical Champions—Administrative and Physician: Across all health centers, executive and clinical champions were foundational for attaining successful implementation. An executive champion is an administrator with overall management responsibilities. These individuals set the stage about what the program is and how it would be impactful. The executive champion typically had budget authority and ensured balance across cross-functional teams.
  In all four health centers a clinical champion provided leadership and motivated teams. The clinical champion made sure that LumineticsCore fit within the existing ecosystem so that the innovation was not construed as another thing demanding resources. The person varied in each of the locations from a Chief Medical Officer, a physician in family medicine, and an endocrinologist. A common theme among all successful clinical champions is that they should have oversight or involvement with the specific clinical locations where LumineticsCore exams are being conducted.
  In our deployments, two of the clinics did not initially involve a clinical champion and consistently experienced lower utilization until one was implemented. At Health Center A after involving a clinical champion, monthly utilization increased from 8 to 95 exams in 3 months. Similar improvements were seen at Health Center C after involving a clinical champion from 16 to 79 exams. In both scenarios the executive champion worked collaboratively with the clinical champion. For smaller health systems, the executive and clinical champion might be the same individual as was the case for Health Center D. Finally it was identified that a lack of a clinical champion often indicated an overall lack of provider adoption. To increase trust and acceptance in the autonomous AI, provider education sessions were given involving the clinical champion detailing workflow updates and providing transparency into the methodology of the AI algorithm.
(II) Underlining Healthcare Center Resources: To deliver on the clinical workflow, all health centers allocated project resources. Table 2 describes the roles of the resources across the key elements of the clinical workflow. All four health centers allocated project resources upfront to appropriately fit into the existing ecosystem. By allocating resources at the beginning of the program deployment, the health centers were able to achieve efficiency and staff satisfaction. Finally, dedicating specific LumineticsCore operators reinforced new skills and helped solidify training in the cases of staff turnover.
(III) Clinical Workflows: Clinical workflows can vary between and within practices. Interjecting additional tasks into existing clinical duties can put clinics behind schedule and increase demands on already resource-thin care team members. All four health centers dealt with the introduction of LumineticsCore by dividing workflow into three primary components illustrated by Figure 2.
  a. Patient Identification (pre-visit): Eligible patient identification is critical for introducing innovation in the front-line setting. Early identification and proactive patient outreach define the health centers with the most success. Health Centers A and C used centralized population health teams or diabetes navigators to run reports identifying eligible patients and subsequently called and scheduled patients in advance of the visit. One of the larger health centers automated patient outreach and notification prior to the visit. The health center additionally implemented self-scheduling with existing visits as part of the patient portal.
    In lower resource centers, such as health center B, eligible patients were identified during the morning huddle. Staff would review the roster of appointments for the day and order the test ahead of time. Health Center D did not have a population health team and instead expanded the role of the Medical Assistant (MA)/Nurse to identify eligible patients for LumineticsCore during vitals. Across all health centers, if a provider identified an eligible patient during the visit, they would place an order and schedule the exam for the end of the visit.
  b. LumineticsCore Exam Capture & Provider Consult (patient visit): The value of point-of-care testing is that it streamlines the referral system, helps with clinical decision-making, and strengthens the patient-provider relationship (4). For instance, some clinics opt to expand roles using nurse navigators (also called diabetes coordinators) to complete exams. In these instances, following the healthcare provider visit, the nurse navigator performs LumineticsCore. At the same time the nurse navigator can provide any related patient education, lab coordination, referrals, and durable medical equipment management (DME), such as insulin pump instructions. Other clinics have an MA capture the exam either just before or after the provider visit.
    When providing results, the decision to allow either the operator or healthcare provider to communicate the findings from LumineticsCore is a decision each clinic uniquely makes. In some cases where the exam is conducted after the healthcare provider consult, the provider would deliver a positive finding to the patient before they left the healthcare center. Others allowed the operator to provide the result.
  c. Timely Referral Triage (post-visit): In all cases, follow up appointments for patients with positive signs of disease were scheduled after the provider visit before leaving the healthcare center. Since three of the four health centers did not have eyecare specialty in the health system, pre-established referral arrangements were arranged prior to LumineticsCore deployment. At least one of the health centers employed a dedicated referral coordinator to ensure follow up through eye care.

**Table 2:** Operational roles and responsibilities in the health center.

Role	Patient identification	Exam capture	Post-exam results/referrals
Population health team	Identification of eligible patients for exam		Documentation of service and care gap closure
MA/Nurse/Front desk	Flagging of eligible patients based on care alerts or identification during huddles or patient rooming	Completes exam with existing diabetes related visit (e.g., office visit, laboratory, diabetes education)	Facilitates referrals to eyecare when needed
Healthcare provider	Identifies patients at time of office visit. Submits order.	Communicates results and develops action plan with patient.	Facilitates referrals to eye care where indicated, completes billing and documentation of care gap closure
Referral coordinator			Facilitates referrals to eyecare when indicated
Technical role (s) including: interface resources, desktop/IT, and security teams	Utilizes and/or enhances existing processes for patient identification	Provides optional integration streamlining orders and result notification to the ordering provider.	Utilizes and/or enhances existing processes to simplify referral communications

## Addressing the core determinants for workflow optimization is an essential part of large-scale adoption of innovation in addition to regulatory clearance and reimbursement

In addition to clinical workflow and related resources, successful adoption of AI in healthcare assumes regulatory clearance to market and sell in addition to reimbursement. There are a number of papers that have been published centering on the ethical considerations for healthcare AI systems to address bias and other concerns with AI, and these thresholds must be met in order to achieve regulatory clearance for promotion and selling in any market (3, 6, 10). As outlined in these papers, the intended use case in clinical flow must be clearly evaluated for FDA clearance, as there can be related impacts on safety, efficacy, ethics, and bias (5).

The general challenges of frontline care clinical workflow have been well documented in the literature (4, 18). Standardization of patient intake, streamlining of care management processes, patient flow coordination, office visit documentation, and use of electronic record systems have been extensively measured and optimized in the primary setting (19–23).

Because healthcare AI is so new, there remains a gap in the literature about challenges and opportunities to embed diagnostic AI into the clinical workflow. Best practices dictate defining an agreed upon definition of success or positive return on investment, having organizational support, and providing appropriate training (24–26). We believe this perspective will provide an initial foundation for considerations of future healthcare AI. These best practices can be generalizable to other autonomous AI systems in front-line care settings, thereby increasing patient access, improving quality of care, and addressing health disparities.
